# Supplementary material for: Rhus vulgaris Meikle fruit-mediated silver nanoparticles: Synthesis, characterization, and potential for sun protection, antioxidant and antibacterial applications
Source: PLoS One. 2025 May 22;20(5):e0324799. doi: 10.1371/journal.pone.0324799 (PMC12097586; doi:10.1371/journal.pone.0324799)
Supplement: Attachment — Submitted filename: Response for reviwer comment.docx [file pone.0324799.s002.docx]

**Response to Reviewers comment**

The authors thank all of the reviewers for their valuable insights and comments on the manuscript With title “*Rhus vulgaris* Meikle Fruit-Mediated Silver Nanoparticles: Synthesis, Characterization, and Potential for Sun Protection, Antioxidant and Antibacterial Applications ”. The reviewers’ comments have been taken into consideration and the manuscript has been revised properly accordingly. The revised manuscript shows the changes with red color text for your purview and kind consideration. The response to each comment has also been given below

**Reviewers' comments**

**Reviewer's Responses to Questions**

1. Is the manuscript technically sounds, and do the data support the conclusions?

The manuscript must describe a technically sound piece of scientific research with data that supports the conclusions. Experiments must have been conducted rigorously, with appropriate controls, replication, and sample sizes. The conclusions must be drawn appropriately based on the data presented.

**Reviewer #1**: Yes

**Response for reviewer 1 comment:** Thank you for your comments

Reviewer #2: Partly

**Response for reviewer 2 comment:** In our study, we have implemented rigorous experimental designs, including appropriate controls, adequate replication, and sufficient sample sizes to ensure the reliability and validity of our findings. Each experiment was carefully designed to minimize bias and variability, allowing us to draw robust conclusions based on the data collected. we made our conclusion is in line with the data

2. Has the statistical analysis been performed appropriately and rigorously?

Reviewer #1: Yes

**Response for reviewer 1 comment:** Thank you for your comments

Reviewer #2: Yes

**Response for reviewer comment #1:** Thank you for your comments

3. Have the authors made all data underlying the findings in their manuscript fully available?

The [PLOS Data policy](http://track.editorialmanager.com/CL0/http:%2F%2Fwww.plosone.org%2Fstatic%2Fpolicies.action%23sharing/1/010f019481553af3-c591257a-f2dd-426d-9b4a-f4a54492eb5f-000000/ukkIoh_8iNmMFXHMgF2uZqw_0nHeuWzlk2hihprg59I=194) requires authors to make all data underlying the findings described in their manuscript fully available without restriction, with rare exception (please refer to the Data Availability Statement in the manuscript PDF file). The data should be provided as part of the manuscript or its supporting information, or deposited to a public repository. For example, in addition to summary statistics, the data points behind means, medians and variance measures should be available. If there are restrictions on publicly sharing data—e.g. participant privacy or use of data from a third party—those must be specified.

Reviewer #1: Yes

**Response for reviewer 1 comment:** Thank you for your comments

Reviewer #2: No

**Response for reviewer comment #2:** Thank you for your comments regarding the PLOS Data policy. we have included all relevant data as part of the manuscript's supporting information. This includes not only summary statistics but also the individual data points that contribute to the means, medians, and variance measures presented in our findings.

4. Is the manuscript presented in an intelligible fashion and written in standard English?

PLOS ONE does not copyedit accepted manuscripts, so the language in submitted articles must be clear, correct, and unambiguous. Any typographical or grammatical errors should be corrected at revision, so please note any specific errors here.

Reviewer #1: Yes

**Response for reviewer 1 comment:** Thank you for your comments

Reviewer #2: No:

**Response for reviewer comment #1:** We have thoroughly reviewed the manuscript to identify and correct any typographical or grammatical errors. We have made revisions to ensure that the language is clear, correct, and unambiguous throughout the text

5. Review Comments to the Author

**(a) Reviewer 1 comments**

Dear Author, the manuscript entitled *Rhus vulgaris* Fruit-Mediated Silver Nanoparticles: Synthesis, Characterization, and Potential for Sun Protection, Antioxidant and Antibacterial Applications was written good but still needs to be revised with the following points.

**Comment #1**: The abstract can be modified and rewritten.

**Response for comment #1:** As per comments corrections are made on the manuscripts line number 19 to 39

**Comment #2**: The significance of *R. vulgaris* can be focused in the first part of the introduction.

**Response for comment #2:** Thank you for your suggestion regarding the placement of information about the significance of *R. vulgaris* in the introduction. As per comments corrections are made on the manuscripts line number 50 to 57

**Comment #3:** The exploration of AgNPs can be effectively written in the introduction with specific biological properties.

**Response for comment #3:** Thank you for your insightful comment regarding the exploration of silver nanoparticles (AgNPs) and their specific biological properties. As per comments corrections are made on the manuscripts line number 76 to 86.

**Comment #4**: Subheadings plant collection and preparation of plant extracts can be merged together and write in simplified manner.

**Response for comment #4:** As per comments corrections are made on line number 95 to 102. We marge three sub topics together and include under subtitle sample collection and preparation. we removed unnecessary additional information.

**Comment #5:** Remove the subheadings of characterization part and merge together.

**Response for comment #5:** As per comments corrections are made on line number 138 to 154. We marge all characterization techniques together and include under subtitle Characterization Techniques. We removed unnecessary additional information

**Comment #6:** Why has the author done only one antioxidant activity?

**Response for comment #6:** Thank you very much for your comments. We used only one antioxidant assay such as DPPH is due to its simplicity, reliability, and relevance to the study's goals, as well as practical considerations regarding resources and time.

**Comment #7:** The optimization part can be emphasized more with some more factors.

**Response for comment #7:** Thank you for your valuable feedback regarding the optimization section of my work. I appreciate your insights and would like to take this opportunity to elaborate on the optimization aspects and the factors that contribute to its synthesis silver nanoparticles. To synthesize silver nanoparticles, several critical parameters must be optimized, including pH, temperature, concentration, and reaction time. Our research work of existing research has highlighted the significant impact of these factors on the synthesis process. Therefore, we have incorporated these optimization parameters into our study to ensure the effective and efficient production of silver nanoparticles

**Comment #8**: The discussion related with AgNPs towards skin related research can be discussed more.

**Response for comment #8:** Thank you very much for the comments. As per comments we include the feedbacks in line number 460 to 472

**Comment #9.** Check for overall grammatical errors and sentence framing.

**Response for comment #9:** We have thoroughly reviewed the manuscript to identify and correct any typographical or grammatical errors. We have made revisions to ensure that the language is clear, correct, and unambiguous throughout the text

**(b) Reviewer #2 comments**

Authors presented “*Rhus vulgaris* Fruit-Mediated Silver Nanoparticles: Synthesis, Characterization, and Potential for Sun Protection, Antioxidant and Antibacterial Applications”, however, it requires more characterization part.

Authors should include and explain the below comments in the revised manuscript

**Comment #1**: Abstract should be rewrite, and should summarize the reason for the work, the most significant results, and the conclusions.

**Response for comment #1:** As per comments corrections are made on line number 19 to 39.

**Comment #2:** In introduction part, several categories of metallic NPs by green synthesis such as Au, Ag, Zno, Pd, TiO2, Cu, etc, have been reported with improved biomimetic attributes. Include these metal and metal oxide NPs with reference, Au (Phytosynthesis of gold nanoparticles using Caesalpinia pulcherrima (peacock flower) flower extract and evaluation of their antimicrobial activities) Ag (<https://doi.org/10.1016/j.matpr.2020.10.048>), ZnO and Cu (<https://doi.org/10.1007/s43630-022-00224-0>), Fe and Pd (DOI: 10.1039/C5CY00099H), etc.

**Response for comment #2:** Thank you for the commons. As per comments corrections are made on line number 77 to 87

**Comment #3**. Synthetic part: Why the authors used *Rhus vulgaris* Fruit extract only instead of other extract?

**Response comment #3:** Thank you very much for the comment. Since there hasn't been any earlier research on using *Rhus vulgaris* fruit extract to synthesize silver nanoparticles, we chose it. *Rhus vulgaris* is also known for its distinct phytochemical profile, traditional therapeutic use, and antioxidant qualities. Our selection of this specific extract is further supported by its accessibility and applicability to the goals of our investigation**.**

**Comment #4:** So many reports already published using the *Rhus vulgaris* Fruit extracts for the synthesis of AgNPs and biomedical application studies?

**Response for comment #4:** Thank you very much for your comment. There are several research works on *Rhus vulgaris* Fruit extracts and its biomedical application. However, as much as we know there is no research works on biomedical application using silver nanoparticle mediated by fruit extract of *Rhus vulgaris* for sun screening application, antioxidant and antibacterial activities

**Comment #5**. UV and IR spectral image should be clear, IR is not clear better to remove it

**Response for comment #5:** As per comments corrections are made. We changed the UV, and IR peaks to be clear figure 3 and 4 (line number 354 and 374)

**Comment #6**: Characterization part: all figures including IR, TEM, should be clear

**Response for comment #6:** As per comments corrections are made. We changed the IR, and TEM peaks to be clear.(line number 449)

**Comment #7:** XRD for AgNPs required with clear hkl values (111, 200, 220, 311, 222 for AgNPs) and explain the hkl values

**Response for comment #7:** As per comments corrections are made. We included hkl vale (111, 200, 220, 311, 222 for AgNPs) in XRD peaks in Figure 6 line 402 and in the text line number 389 to 392

**Comment #8**: Single particle or HRTEM should be included with EDAX

**Response for comment #8:** Thank you very much for nice comments. Because of the instruments available, for particle size determination, we utilized transmission electron microscopy (TEM), and to detect the presence of silver (Ag) metal, we employed UV-Vis spectroscopy due to the availability of instruments. In our future work we will include this nice comments

**Comment #9**: Author should explain how the average size of AgNPs is 10 nm to 20 nm?

**Response for comment #9:** Thank you very much for the comments. Using TEM image and imaje software the average size nanoparticle was calculated. This is indicated in line number 440 to 441

**Comment #10**: In addition, the lattice fringe spacings or D spacing hkl should be included

**Response for comment #10:** As per comments corrections are made on line number 396 to 397

**Comment #11**: For antioxidant activity: author performed DPPH, what about ABTS, and NOx radical scavenging analyses? Compare and refer (<https://doi.org/10.2147/IJN.S210517>)

**Response for comment #11:** Thank you very much for your comments. We used only one antioxidant assay such as DPPH is due to its simplicity, reliability, and relevance to the study's goals, as well as practical considerations regarding resources and time. As per comments corrections are made on line number 546 to 552

**Comment #**12. For antibacterial activity refer (DOI 10.1088/2053-1591/ad1357)

**Response for comment #12:** Thank you for your comments. As per comments corrections are made in line number 635 to 641

**Comment #**13. Compare the current work with previously published reports for AgNPs in separate table

**Response for comment #13:** As per corrections are made. We added new table (Table 3) that compare current work with other result in line number 404 to 433

**Comment #**14. Conclusions should be rewrite

**Response for comment #14:** As per comments corrections are made on line number 656-674

**Comment #**15. Major English edition is required for whole manuscript

**Response for comment #15:** We checked the English edition throughout the paper
